# Yellow Fever Commission

**Published:** 1886-06

**Authors:** 


YELLOW FEVER COMMISSION.

Congress has passed the bill providing for a commission of three physicians to visit Brazil and other foreign tropical countries for the purpose of studying the alleged inoculative protection process of Friere and Carmona. Uncle Sam once investigated Condurango, in Brazil.

